# Physical Confirmation and Mapping of Overlapping Rat Mammary Carcinoma Susceptibility QTLs, *Mcs2* and *Mcs6*

**DOI:** 10.1371/journal.pone.0019891

**Published:** 2011-05-18

**Authors:** Jennifer Sanders, Jill D. Haag, David J. Samuelson

**Affiliations:** 1 Department of Biochemistry and Molecular Biology, University of Louisville School of Medicine, Louisville, Kentucky, United States of America; 2 McArdle Laboratory for Cancer Research, University of Wisconsin-Madison, Madison, Wisconsin, United States of America; 3 James Graham Brown Cancer Center, University of Louisville School of Medicine, Louisville, Kentucky, United States of America; 4 Center for Genetics and Molecular Medicine, University of Louisville School of Medicine, Louisville, Kentucky, United States of America; 5 Center for Environmental Genomics and Integrative Biology, University of Louisville School of Medicine, Louisville, Kentucky, United States of America; Ohio State University Medical Center, United States of America

## Abstract

Only a portion of the estimated heritability of breast cancer susceptibility has been explained by individual loci. Comparative genetic approaches that first use an experimental organism to map susceptibility QTLs are unbiased methods to identify human orthologs to target in human population-based genetic association studies. Here, overlapping rat *mammary carcinoma susceptibility* (*Mcs*) predicted QTLs, *Mcs6* and *Mcs2,* were physically confirmed and mapped to identify the human orthologous region. To physically confirm *Mcs6* and *Mcs2,* congenic lines were established using the Wistar-Furth (WF) rat strain, which is susceptible to developing mammary carcinomas, as the recipient (genetic background) and either Wistar-Kyoto (WKy, *Mcs6*) or Copenhagen (COP, *Mcs2*), which are resistant, as donor strains. By comparing Mcs phenotypes of WF.WKy congenic lines with distinct segments of WKy chromosome *7* we physically confirmed and mapped *Mcs6* to ∼33 Mb between markers *D7Rat171* and *gUwm64-3*. The predicted *Mcs2* QTL was also physically confirmed using segments of COP chromosome *7* introgressed into a susceptible WF background. The *Mcs6* and *Mcs2* overlapping genomic regions contain multiple annotated genes, but none have a clear or well established link to breast cancer susceptibility. *Igf1* and *Socs2* are two of multiple potential candidate genes in *Mcs6.* The human genomic region orthologous to rat *Mcs6* is on chromosome *12* from base positions *71,270,266 to 105,502,699*. This region has not shown a genome-wide significant association to breast cancer risk in pun studies of breast cancer susceptibility.

## Introduction

Breast cancer susceptibility is a complex trait controlled by genetic, epigenetic, and environmental components. Susceptibility to breast cancer is comprised of low, moderate, and high penetrance risk alleles. Linkage analysis in high incidence families and population-based genetic association studies using cases and controls have identified breast cancer risk alleles; however, a large portion of the estimated heritability in breast cancer susceptibility remains to be explained [1], [2]. Laboratory rats (*Rattus norvegicus*) have been used to identify human breast cancer risk alleles using comparative genetics [3]. To identify loci for comparative studies, rat strains that exhibited phenotypic variation in susceptibility to chemical and hormone induced mammary carcinogenesis have been used to predict *mammary carcinoma susceptibility* (*Mcs*) and *estrogen-induced mammary cancer* QTLs [4]. Rats were chosen to identify *Mcs* QTLs because chemically induced rat mammary carcinomas have similar histopathology to human breast carcinomas [5]. The Wistar Furth (WF) rat strain is susceptible to developing mammary carcinomas, while the Wistar Kyoto (WKy) and Copenhagen (COP) rat strains are resistant [6], [7]. A QTL linkage analysis of rat Mcs in females from [WF x WKY]_F1_ x WF backcrosses predicted four *Mcs* QTLs named *Mcs5, Mcs6, Mcs7,* and *Mcs8* [7]. The *Mcs6* QTL displayed a peak linkage at marker *D7Mit28* and significant linkage was found between markers *D7Mit28* and *D7Rat45.* The predicted *Mcs6* QTL region overlapped with *Mcs2,* a *Mcs* QTL predicted using the mammary cancer resistant Copenhagen (COP) rat strain [6]. The peak LOD score for the predicted *Mcs2* QTL was at genetic marker *D7Uwm11.* Here, we physically confirm both *Mcs2* and *Mcs6* QTLs, and map *Mcs6* to an approximately 33 Mb region of rat chromosome *7*, which is orthologous to a region on human chromosome *12*.

## Results

### Congenics physically confirm and map *Mcs6* to a 33Mb region on rat chromosome *7*

**Figure 1:**
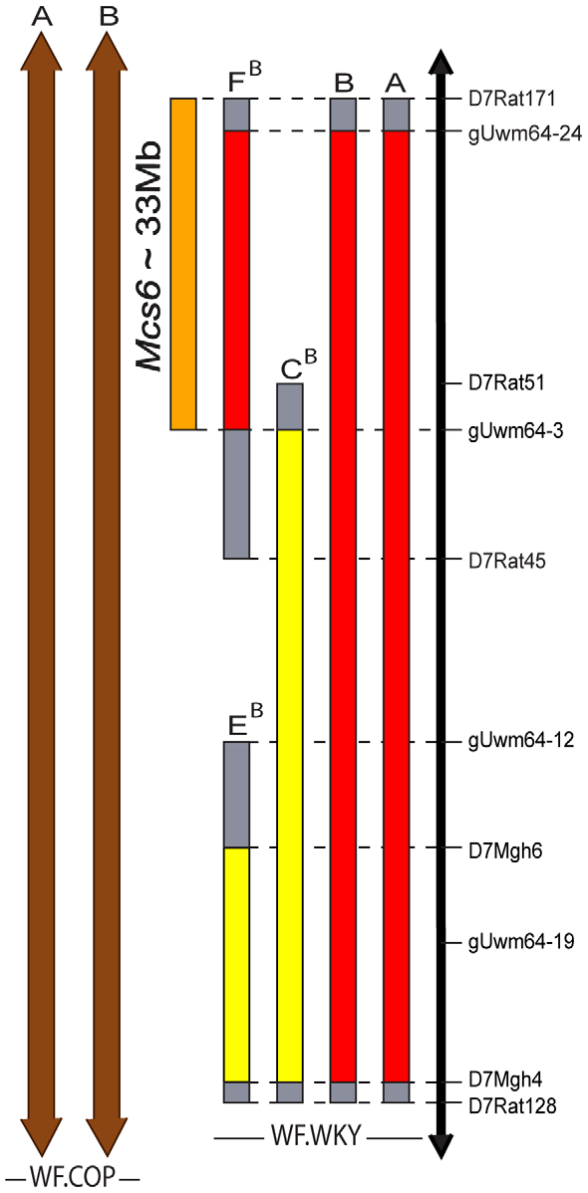
Congenic lines used to physically confirm *Mcs6* and *Mcs2* QTLs and map the *Mcs6* locus to ∼33 Mb on rat chromosome *7*. Informative genetic markers between WF and WKy rat strains used in the genotyping of WF.WKy chromosome *7* lines are shown on the right of the y-axis, which represents the region of rat chromosome *7* that contains *Mcs6*. Congenic lines were established using genetic marker genotype selection and backcrossing to WF homozygous females, which was followed by brother-sister mating to fix homozygous congenic lines in the chromosomal segments shown. Superscript letters indicate which line the respective superscripted line was derived from. WF.WKy chromosome *7* lines A, B, and F (red bars) all showed a statistically significant decrease in mean tumor number compared to WF control females (respective Mann-Whitney *post hoc* p-values were = 0.0012, 0.0007, and 0.0001), while lines C and E (yellow bars) were not significantly different from the susceptible WF phenotype (Mann-Whitney *post hoc* p-values = 0.7521and 0.7893, respectively). The gray bars at the ends of congenic line segments denote regions of unknown genotype. WF.COP chromosome *7 Mcs2* congenic lines A and B that physically confirm the *Mcs2* QTL and overlap the *Mcs6* QTL are shown with brown bars that represent the segment of COP chromosome *7* contained in each line. The arrows at the end of *Mcs2* congenic line segments indicate that the COP chromosome *7* segment continues off this map. WF.COP chromosome *7 Mcs2* lines A and B developed significantly less mammary carcinomas than WF control females (Mann-Whitney *post hoc* p-values <0.0001).

Figure 1 depicts the WF.WKy congenic lines used to physically confirm and map the predicted *Mcs6* QTL to a location on rat chromosome *7* between markers *D7Rat171* and *gUwm64-3*. Table 1 summarizes mammary carcinoma susceptibility (Mcs) phenotypes from lines shown in Figure 1. Respectively, WF.WKy lines A, B, and F had mammary carcinoma multiplicity phenotypes (mean ± SD carcinomas *per* rat) of 3.5±2.5, 3.9±2.6, and 3.4±2.1. These lines developed significantly less mammary carcinomas compared to susceptible WF control animals, which had a mean ± SD of 7.3±3.6 carcinomas *per* rat (Mann-Whitney test *post hoc* p-values = 0.0012, 0.0007, and 0.0001). Females from WF.WKy lines A, B, and F had similar mammary carcinoma multiplicity phenotypes when compared to each other (Kruskal-Wallis test p-value = 0.8171). WF.WKy line C had a mean ± SD of 8.0±4.1 mammary carcinomas *per* rat, and line E had a mean ± SD of 7.2±3.0. Neither line C nor line E was significantly different from WF control animals (Mann-Whitney test *post hoc* p-values = 0.7521 and 0.7893, respectively). Decreased susceptibility WF.WKy lines A, B, and F overlapped maximally from markers *D7Rat171* to *D7Rat45,* and minimally from *gUwm64-24* to *gUwm64-3.* We conclude that these lines physically confirm *Mcs6* as an independently acting *Mcs* QTL.

**Table 1:** Summary of mammary carcinoma multiplicity phenotypes from WF.WKy and WF.COP rat chromosome *7* congenic lines used to map *Mcs6* and *Mcs2*.

	WF.WKy Congenic Line					WF.COP Congenic Line		
	A	B	C	E	F	A	B	WF
**Congenic Region** 1 ***Marker/Marker***	*D7Rat171/D7Rat128*	*D7Rat171/D7Rat128*	*D7Rat51/D7Rat128*	*gUwm64-12/D7Rat128*	*D7Rat171/D7Rat45*	*D7rat39/D7Uwm12*	*D7rat39/D7Uwm12*	-
**Mean (SD) Mammary Carcinomas ** ***per*** ** Rat**	3.5 (2.5)	3.9 (2.6)	8.0 (4.1)	7.2 (3.0)	3.4 (2.1)	2.4 (1.6)	2.0 (1.5)	7.3 (3.6)
**N**	17	32	23	43	28	16	16	19
**p-value** 2	0.0012	0.0007	0.7521	0.7893	0.0001	<0.0001	<0.0001	-

1 Markers spanning the maximal WKy or COP Chr *7* segment that was introgressed onto a susceptible WF genetic background are given.

2 p-values are from Mann-Whitney nonparametric *post hoc* tests comparing each congenic line to the WF phenotype after a statistically significant Kruskal-Wallis test with a p-value <0.0001.

WF.WKy line C, which was not significantly different from susceptible WF control animals, overlapped the distal end of the *Mcs6* region demarked by lines A, B, and F from markers *gUwm64-3* to *D7Rat128.* This ruled out that this region contained *Mcs6* or independently acting susceptibility QTLs. The susceptible phenotype of line E, which partially overlapped susceptible line C, confirmed that the region from *gUwm64-12* to *D7Rat128* did not contain an independently acting *Mcs* QTL. When considered together, our results from WF.WKy congenic lines delimited *Mcs6* to a 33 Mb region of rat chromosome *7* from markers *D7Rat171* to *gUwm64-3.*

### *Mcs6* overlaps *Mcs2* a Copenhagen rat QTL associated with mammary cancer susceptibility

*Mcs2* is a Copenhagen (COP) rat *Mcs* QTL that was also predicted to reside on chromosome *7* [6]. The original QTL scans of *Mcs2* (COP) and *Mcs6* (WKy) predicted regions of significant linkage on rat chromosome *7* that overlapped from markers *D7Mgh15* to *D7Mgh10* [7]. We physically confirmed *Mcs2* using WF.COP congenic lines A and B that each contained a COP segment of rat chromosome *7* from markers *D7rat39* to *D7Uwm12* (Figure 1). These markers corresponded to rat chromosome *7* base positions 4,936,704-86,028,057. WF.COP lines A and B had similar mammary carcinoma multiplicity phenotypes of 2.4±1.6 and 2.0±1.5 mean ± SD carcinomas *per* rat (Table 1, Mann-Whitney test p-value 0.4510). Both lines were significantly less than the WF susceptible phenotype (Mann-Whitney *post hoc* test p-values <0.0001). The confirmed *Mcs2* QTL overlapped the delimited *Mcs6* QTL between markers *D7Rat171* and *gUwm64-3* (Figure 1). The pooled phenotypes of *Mcs2* (WF.COP lines A and B) and *Mcs6* (WF.WKy lines A, B, and F) females were, respectively, 2.2±1.5 and 3.6±2.4 mean ± SD mammary carcinomas *per* rat. These phenotypic values were statistically different (Mann-Whitney test p-value = 0.0030) suggesting that the *Mcs2* COP allele may have additional genetic variation contributing to mammary carcinoma resistance.

### The human ortholog to rat *Mcs6* is on chromosome *12*

Using the UCSC rat and human genome browsers (genome.ucsc.edu) we determined that the *Mcs6* QTL from markers *D7Rat171* to *gUwm64-3* (chr*7*:22,382,725-55,364,398) aligns to a contiguous 34.2 Mb region of human chromosome *12* from base positions 71,299,117 to 105,502,699, which was inverted with respect to the rat genome assembly. Within these orthologous regions 37.7% of the bases over 99.9% of the span were identical. Rat genomic sequence flanking *Mcs6* aligned to human chromosome *12* up to rat chromosome *7* base positions 22,310,240 on one flank and *55*,442,351 on the other flank. Moving out past these syntenic regions of rat chromosome *7* and human chromosome *12* resulted in rat genomic sequence that aligned to human chromosome *16* on one flank and chromosome *X* on the other.

**Figure 2:**
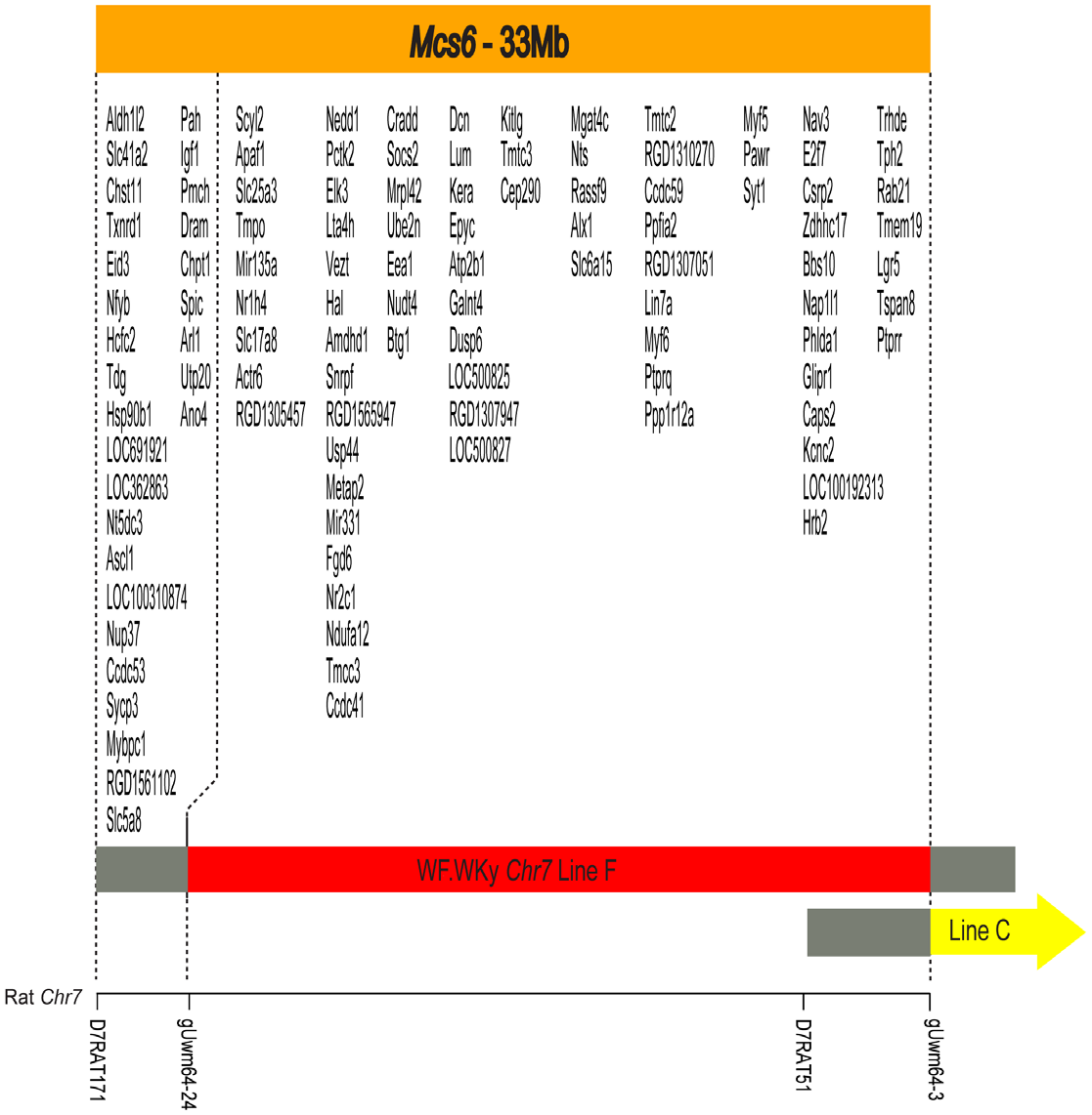
Transcript map of the delimited rat *Mcs6* QTL. Map of the *Mcs6* locus showing known and predicted transcripts in the *Mcs6* region that were annotated on the UCSC Rat Nov. 2004 (Baylor3.4/rn4) Genome Browser. The x-axis represents rat chromosome *7* from genetic markers *D7Rat171* to *gUwm64-3;* a rat chromosome *7* region from bp 22,382,725-55,364,398 that contains *Mcs6.* The dashed vertical lines demark the respective maximal and minimal rat genomic regions that contain *Mcs6.* Transcripts are shown by NCBI gene symbol at approximate genomic positions within *Mcs6*. Genetic markers within the region are shown as labeled tick marks on the x-axis. WF.WKy chromosome *7* lines F (red horizontal bar, resistant Mcs phenotype) and C (yellow horizontal bar, susceptible Mcs phenotype) are included to serve as reference points. The gray horizontal bars at the ends of congenic lines denote a segment of unknown genotype for the respective line.

Figure 2 shows annotated genes in the *Mcs6* QTL between markers *D7Rat171* and *gUwm64-3.* These genes were on the UCSC rat November 2004 assembly (build 3.4/rn4). Not including pseudogenes, this region contained 111 annotated genes including two microRNAs (*Mir135* and *Mir331*). There were 137 annotated genes found in *Mcs6* using the Rat Genome Database (rgd.mcw.edu), and 215 found using the Ensembl Genome Browser (uswest.ensembl.org) rat database. Independent searches of *Mcs6* potential candidate genes using gene ontology terms limited to mammary, hormone, estradiol, or DNA repair resulted in the following gene list: *Socs2, Igf1, Nr1h4, Osbpl8, Nr2c1, Pmch, Stab2, Nts, Lgr5, Btg1, Lta4h,* and *Ube2n.* All genes located in rat *Mcs6* were also annotated in the human orthologous region. There were a total of 243 genes listed in the human orthologous region when the UCSC human genome browser was queried and 226 genes when the Ensembl browser was used.

We queried the Oncomine 4.4 database (www.oncomine.org, Compendia Bioscience) to identify *Mcs6* human orthologs that may have been reported as differentially expressed between non-diseased breast tissue and ductal breast carcinomas. A list of the studies included can be found in References S1 (S1-S7). Results from this search are presented in Table S1. There were twenty-nine transcripts reported with p-values <0.05, and six of these had p-values <0.001 *Mcs6* human orthologs with p-values <0.001 were *ALX1, DUSP6, EPYC, PPP1R12A, TXNRD1,* and *ZDHHC17.*

To determine if genetic variation in the *Mcs6* orthologous human genomic region from chromosome *12:71,270,266-105,502,699* might be associated with breast cancer susceptibility we searched the NCBI dbGAP database using the association results browser set to “breast neoplasms” and chromosome 12 (www.ncbi.nlm.nih.gov/gap). We also searched published breast cancer risk GWA studies for positive associations in this region. A list of the association studies included can be found in References S1 (S8-S23). While five human SNPs were identified that potentially associated with breast cancer susceptibility, tested genetic variants did not reach a genome-wide significance level of P< 10^-7^ proposed by Thomas *et al.* [8]. A summary of human SNPs in the *Mcs6* orthologous region that potentially associate with breast cancer risk can be found in Table S2.

## Discussion

Both mammary cancer susceptibility QTL mapping in rats and low-penetrance breast cancer risk associated allele identification in humans are in early stages of discovery. Results of human population-based and rat genetic studies completed to date indicate that a majority of breast cancer risk alleles are low-penetrance, and pathways in addition to DNA repair and hormonal regulation control susceptibility. Furthermore, a majority of the risk associated polymorphisms that have been identified reside in non-protein coding DNA. Given the commonalities of low-penetrance, novel pathways, and non-protein-coding risk alleles, it is possible that there is some degree of overlap in susceptibility loci between these two organisms. There may be shared susceptibility loci that can be more easily identified in rats than by current human GWAS methods. If this is the case, resolving rat mammary carcinoma susceptibility QTLs to single loci so that the human orthologs can be extensively tested in human population-based genome-targeted association studies may continue to be fruitful. This was the case for Gould and colleagues who used rat *Mcs5a* to successfully target the orthologous human genomic region to identify two breast cancer susceptibility alleles on human chromosome *9* [3]. Quan *et al.* have shown that rodent susceptibility QTLs identified for a specific tumor type may also be useful to identify common susceptibility alleles for other tumor types [9]. Thus, the amount of overlap between rodent and human common disease susceptibility loci may be appreciable.

Here, predicted rat mammary carcinoma susceptibility QTLs *Mcs6* and *Mcs2* were physically confirmed using congenics. In our study *Mcs2* and *Mcs6*, respectively, reduced the mammary carcinoma susceptibility phenotype of the WF strain by 70 and 50% when homozygous. In the original QTL studies, one *Mcs2* COP or one *Mcs6* WKy allele was predicted to reduce tumor multiplicity by 43% or 40%, respectively [6], [7]. While those studies were not completed using congenics, and we have not tested heterozygous females, comparing the results of tumor numbers of homozygous *Mcs2* and *Mcs6* congenics with heterozygous females from the previous studies suggests there may be some degree of dominance from the WF allele at this locus. However, these differences may also be explained by background COP and WKy alleles that would have been present in females used for the QTL linkage studies. The stronger effect of the *Mcs2* COP allele compared to the *Mcs6* WKy allele may indicate that the Copenhagen strain has additional genetic variation within the region that contributes to reducing the Mcs phenotype.

In this study *Mcs6* was mapped to a 33 Mb region from markers *D7Rat171* to *gUwm64-3*, which corresponds to rat chromosome 7 base positions 22,382,725-55,364,398. The peak LOD scores for genetic markers in the predicted *Mcs6* and *Mcs2* QTLs were at *D7Mit28* and *D7Uwm11*, respectively [6], [7]. Both peak QTL markers fall within the narrowed interval confirmed here with congenics. *D7Mit28* is at base position Chr*7:*29,204,226, and *D7Uwm11* is at base position Chr*7:*50,160,469. This supports using QTL linkage analysis of rat complex phenotypes to identify regions of the genome on which to focus targeted human genome association studies.

Alignment of the rat *Mcs6* QTL region to the human genome reference sequence revealed that the human orthologous region is entirely positioned on 34.2 Mb of chromosome *12,* and is inverted compared to rat *Mcs6*. To our knowledge no polymorphisms in the human genome region orthologous to rat *Mcs6* have shown a genome-wide significant association to breast cancer risk in a GWAS. An intriguing potential candidate gene in *Mcs6* is *insulin-like growth factor 1* (*Igf1*). Human candidate-gene association studies that have tested *IGF1* locus polymorphisms reported mixed results ranging from significant low-penetrance to null associations between *IGF1* alleles and breast cancer risk [10]–[15]. Increased IGF1 levels have been associated with breast cancer and increased breast cancer susceptibility [16], [17]. Common genetic variation in *IGF1* has been associated with circulating levels of IGF1 in a population-based study [15]. Other *Mcs6* potential candidates have human orthologs that have been associated with cancer or other human diseases. *SLC5A8* and *GLIPR1* are predicted tumor repressor genes [18], [19]. *METAP2* is predicted to be anti-angiogenic [20]. A human genome-wide linkage analysis of posterior amorphous corneal dystrophy identified a region of human chromosome *12* that is orthologous to a *Mcs6* region that contains genes *DCN, LUM, KERA*, and *EPYC* [21]. *PCTK2* has been shown to be upregulated in sporadic breast cancer biopsies in women of Mexican ancestry [22]. *Suppressor of cytokine signaling 2* (*Socs2*) is also a promising potential candidate gene in *Mcs6.* Higher SOCS2 levels have been associated with better breast cancer prognosis [23]–[25]. *Socs2* is an important mediator of prolactin regulated mammary gland development in the mouse [26]. *SOCS2* is upregulated by 17β-estradiol in cancer cell lines [27], and *SOCS2* expression was found to be higher in breast carcinomas compared to non-diseased breast tissue [28].

*Mcs6* human orthologous transcripts that have been reported as differentially expressed between non-diseased breast tissue and ductal carcinoma tissue encode for two phosphatases (DUSP6, PPP1R12A), a proteoglycan (EPYC), a redox regulator (TXNRD1), and two uncharacterized proteins (ALX1, ZDHHC17). These findings suggest that the rat *Mcs2* and *Mcs6* QTLs control potentially novel mechanisms contributing to mammary cancer susceptibility that could translate to breast cancer.

Human genetic variation in the *Mcs6* orthologous genomic region has been reported to be associated with breast cancer susceptibility, but tested polymorphisms have not reached genome-wide level significance. This highlights the importance of this region to breast cancer genetics. It suggests, as many have, that Bonferroni correction based methods may be too stringent to control for multiple comparison testing in human population-based association studies. The confirmed rat *Mcs2* and *Mcs6* QTLs support that some or all of the potential human breast cancer risk associations are true positives and warrant further investigation.

We conclude that the human genomic region on chromosome *12* from base positions *71,270,266 - 105,502,699* is orthologous to rat *Mcs6*. This region may be a good target for extensive population-based genetic association studies to determine if humans have a concordant breast cancer susceptibility allele. Rat *Mcs6* and *Mcs2* congenic lines will be useful to study breast cancer genetic susceptibility mechanisms.

## Materials and Methods

### Congenic Breeding and Genotyping

Congenic lines were established using previously described methods [29] and are defined as rats with specified WKy chromosome *7* segments from the predicted *Mcs6* QTL or the specified COP chromosome *7* segments from the predicted *Mcs2* QTL introgressed into a WF genetic background. Rats were housed in an AALACC approved facility and protocols were approved by a University of Louisville institutional animal care and use committee under protocol number 07118. Genotyping was performed at the markers shown in Figure 1 according to methods previously described [29]. Primer and sequence information for most genetic markers used is available at the Rat Genome Database (rgd.mcw.edu) [30]. The following primer sequence pairs are for genetic markers that were submitted to the NCBI probe database, but may not yet be publicly available: *gUwm64-24,* 5′-TGTCCTCTCATACCACTTCC-3′, 5′-CGGTGGTCATACTCTGAAT-3′; *gUwm64-3*, 5′-AACAGTTCTCCTTTCCCTTC-3′, 5′-TCTCTTTGCCAGTCTGTTTT-3′, *gUwm64-12,* 5′-CTGGTCATTCAACAGGAAAT-3′, 5′-CCTATCCCTGTTCTTCCTCT-3′; and *gUwm64-19,* 5′-AACAATAGGCACAGTGAT-3′, 5′-ATGCTGTCAGAACGGTAAGT-3′.

### Phenotyping

Female congenic and WF (Harlan) rats at 50-55 days of age were treated with a single dose of 7,12-Dimethylbenz(a)anthracene (DMBA, ACROS Organics, Fisher Scientific) in sesame oil by gastric intubation at 65 mg DMBA/kg body mass. Mammary carcinomas ≥3×3 mm^2^ were counted at 15 weeks following DMBA treatment. Mammary carcinoma multiplicity data were analyzed to compare congenic line phenotypes to susceptible WF control females. WF.WKy chromosome *7* congenic phenotypes for each line were from the following generations: line A, N11F2 and N11F4 (total n = 17); line B, N11F2 and N11F3 (total n = 32); line C, N14F2, N14F3, and N14F4 (total n = 23); line E, N14F2 and N14F4 (total n = 43); and line F, N14F3 and N14F4 (total n = 28). WF.COP chromosome *7* congenic phenotypes were from generations: line A, N10F5 (n = 16); and line B, N11F3 and N11F4 (total n = 16).

### Statistical analysis

To compare mammary carcinoma susceptibility phenotypes, mammary carcinoma multiplicity data were analyzed using Statview (SAS Institute). Mammary carcinoma multiplicity phenotypes for more than two groups were compared using the Kruskal-Wallis nonparametric test. If this test was statistically significant (p value <0.05) multiple Mann-Whitney nonparametric tests were performed by comparing each congenic line phenotype to the susceptible WF control group. To correct for multiple comparisons a p-value ≤0.007 (α = 0.05/7 comparisons) was required for statistical significance. For analysis of two groups, Mann-Whitney nonparametric tests were performed and p-values ≤0.05 were required for statistical significance.

### Bioinformatics

Identification of the human orthologous region and transcripts mapping to human and rat regions of interest were performed using the RGD, UCSC, and Ensembl genome browsers [30]-[32]. The genome assemblies used were *Homo sapiens* version GRCh37/hg19 and *Rattus norvegicus* version 3.4/rn4. The percent sequence identity between human and rat was determined using the “Convert” option at the UCSC Genome Browser. The gene ontology search was completed by downloading from the Rat Genome Database gene ontology terms for genes annotated in *Mcs6* and searching this database by terms: “mammary”, “hormone”, “estradiol”, or “DNA repair”.

## Supporting Information

Table S1 Results of a query using Oncomine to identify human *Mcs6* orthologous transcripts that have been shown to be differentially expressed between non-diseased and ductal carcinoma breast tissues.
(DOCX)

Table S2 Human polymorphisms located in the *Mcs6* orthologous region that have not reached genome-wide significance, but are potentially associated with breast cancer susceptibility.
(DOCX)

References S1 Citations for supplemental references.
(DOCX)
