# Supplementary material for: Physical Confirmation and Mapping of Overlapping Rat Mammary Carcinoma Susceptibility QTLs, Mcs2 and Mcs6
Source: PLoS One. 2011 May 18;6(5):e19891. doi: 10.1371/journal.pone.0019891 (PMC3097214; doi:10.1371/journal.pone.0019891)
Supplement: Table S1 — Results of a query using Oncomine to identify human Mcs6 orthologous transcripts that have been shown to be differentially expressed between non-diseased and ductal carcinoma breast tissues. (DOCX) [file pone.0019891.s001.docx]

| **Table S1. *Mcs6* Human Ortholog Gene Expression Compared Between**  **Non-Diseased Breast Tissue and Ductal Breast Carcinoma Using Publicly Available Databases.** | | | |
| --- | --- | --- | --- |
| **Gene Name** | **Expression** | **P-value** | **Studies Reporting Significant Expression Differences** |
| *ACTR6* | no difference |  |  |
| *ALDH1L2* | no difference |  |  |
| *ALX1* | increased | 1.67x10^-4^ | Zhao *et al.*  Sørlie *et al.* (2001)  Richardson *et al.* |
| *AMDHD1* | increased | 0.012 | Richardson *et al.* |
| *ANO4* | no difference |  |  |
| *APAF1* | no difference |  |  |
| *ARL1* | increased | 0.002 | Richardson *et al.* |
| *ASCL1* | no difference |  |  |
| *ATP2B1* | no difference |  |  |
| *BBS10* | no difference |  |  |
| *BTG1* | no difference |  |  |
| *CAPS2* | no difference |  |  |
| *CCDC41* | no difference |  |  |
| *CCDC53* | no difference |  |  |
| *CCDC59* | no difference |  |  |
| *CEP290* | no difference |  |  |
| *CHPT1* | decreased | 0.011 | Perou *et al.*  Richardson *et al.*  Sørlie *et al.* (2001)  Sørlie *et al.* (2003)  Turashvili *et al.*  Zhao *et al.* |
| *CHST1* | No difference |  |  |
| *CRADD* | decreased | 0.037 | Richardson *et al.* |
| *CSRP2* | no difference |  |  |
| *DCN* | no difference |  |  |
| *DRAM* | no difference |  |  |
| *DUSP6* | decreased | 2.92x10^-5^ | Perou *et al.*  Richardson *et al.*  Sørlie *et al.* (2001)  Sørlie *et al.* (2003)  Zhao *et al.* |
| *E2F7* | no difference |  |  |
| *EEA1* | no difference |  |  |
| *EID3* | increased | 0.005 | Richardson *et al.*  Turashvili *et al.* |
| *ELK3* | no difference |  |  |
| *EPYC* | increased | 3.76x10^-5^ | Perou *et al.*  Richardson *et al.*  Sørlie *et al.* (2001)  Sørlie *et al.* (2003)  Zhao *et al.* |
| *FGD6* | no difference |  |  |
| *GALNT4* | no difference |  |  |
| *GLIPR1* | no difference |  |  |
| **Table S1 *Continued*. *Mcs6* Human Ortholog Gene Expression Compared Between Non-Diseased Breast Tissue and Ductal Breast Carcinoma Using Publicly Available Databases** | | | |
| **Gene Name** | **Expression** | **P-value** | **Studies Reporting Significant Expression Differences** |
| *HAL* | no difference |  |  |
| *HCFC2* | no difference |  |  |
| *HRB2* | increased | 0.017 | Richardson *et al.*  Zhao *et al.* |
| *HSP90B1* | increased | 0.005 | Perou *et al.*  Richardson *et al.*  Sørlie *et al.* (2001)  Sørlie *et al.* (2003)  Zhao *et al.* |
| *IGF1* | decreased | 0.028 | Perou *et al.*  Richardson *et al.*  Sørlie *et al.* (2001)  Sørlie *et al.* (2003)  Zhao *et al.* |
| *KCNC2* | no difference |  |  |
| *KERA* | decreased | 0.048 | Richardson *et al.* |
| *KITLG* | increased | 0.003 | Richardson *et al.* |
| *LGR5* | no difference |  |  |
| *LIN7A* | no difference |  |  |
| *LOC100192313* | nr |  |  |
| *LOC100310874* | nr |  |  |
| *LOC362863* | nr |  |  |
| *LOC500825* | nr |  |  |
| *LOC500827* | nr |  |  |
| *LOC691921* | nr |  |  |
| *LTA4H* | no difference |  |  |
| *LUM* | decreased | 0.032 | Richardson *et al.* |
| *METAP2* | increased | 0.002 | Richardson *et al.* |
| *MGAT4C* | no difference |  |  |
| *MIR135A* | nr |  |  |
| *MIR331* | nr |  |  |
| *MRPL42* | increased | 0.021 | Perou *et al.*  Richardson *et al.*  Turashvili *et al.*  Zhao *et al.* |
| *MYBPC1* | decreased | 0.002 | Richardson *et al.*  Turashvili *et al.* |
| *MYF5* | no difference |  |  |
| *MYF6* | no difference |  |  |
| *NAP1L1* | no difference |  |  |
| *NAV3* | decreased | 0.014 | Radvanyi *et al.*  Richardson *et al.*  Turashvili *et al.*  Zhao *et al.* |
| *NDUFA12* | no difference |  |  |
| *NEDD1* | increased | 0.021 | Radvanyi *et al.*  Richardson *et al.*  Zhao *et al.* |
| *NFYB* | increased | 0.044 | Turashvili *et al.* |
| **Table S1 *Continued*. *Mcs6* Human Ortholog Gene Expression Compared Between Non-Diseased Breast Tissue and Ductal Breast Carcinoma Using Publicly Available Databases** | | | |
| **Gene Name** | **Expression** | **P-value** | **Studies Reporting Significant Expression Differences** |
| *NR1H4* | no difference |  |  |
| *NR2C1* | no difference |  |  |
| *NT5DC3* | no difference |  |  |
| *NTS* | no difference |  |  |
| *NUDT4* | no difference |  |  |
| *NUP37* | no difference |  |  |
| *PAH* | no difference |  |  |
| *PAWR* | no difference |  |  |
| *PCTK2* | no difference |  |  |
| *PHLDA1* | no difference |  |  |
| *PMCH* | increased | 0.002 | Radvanyi *et al.*  Richardson *et al.* |
| *PPFIA2* | no difference |  |  |
| *PPP1R12A* | decreased | 2.90x10^-4^ | Richardson *et al.*  Sørlie *et al.* (2001)  Sørlie *et al.* (2003) |
| *PTPRQ* | nr |  |  |
| *PTPRR* | no difference |  |  |
| *RAB21* | no difference |  |  |
| *RASSF9* | no difference |  |  |
| *RGD1205457* | nr |  |  |
| *RGD1307051* | nr |  |  |
| *RGD1307947* | nr |  |  |
| *RGD1310270* | nr |  |  |
| *RGD1561102* | nr |  |  |
| *RGD1565947* | nr |  |  |
| *SCYL2* | no difference |  |  |
| *SLC17A8* | no difference |  |  |
| *SLC25A3* | no difference |  |  |
| *SLC41A2* | no difference |  |  |
| *SLC5A8* | no difference |  |  |
| *SLC6A15* | increased  decreased | 0.037  0.041**^1^** | Richardson *et al.*  Sørlie *et al.* (2001)  Sørlie *et al.* (2003)  Zhao *et al.* |
| *SNRPF* | increased | 0.010 | Perou *et al.*  Radvanyi *et al.*  Sørlie *et al.* (2001)  Sørlie *et al.* (2003) |
| *SOCS2* | no difference |  |  |
| *SPIC* | no difference |  |  |
| *SYCP3* | no difference |  |  |
| *SYT1* | no difference |  |  |
| *TDG* | increased | 0.040 | Richardson *et al.*  Sørlie *et al.* (2001)  Sørlie *et al.* (2003)  Zhao *et al.* |
| *TMCC3* | no difference |  |  |
| *TMEM19* | no difference |  |  |
| **Table S1 *Continued*. *Mcs6* Human Ortholog Gene Expression Compared Between Non-Diseased Breast Tissue and Ductal Breast Carcinoma Using Publicly Available Databases** | | | |
| **Gene Name** | **Expression** | **P-value** | **Studies Reporting Significant Expression Differences** |
| *TMPO* | no difference |  |  |
| *TMTC2* | no difference |  |  |
| *TMTC3* | no difference |  |  |
| *TPH2* | no difference |  |  |
| *TRHDE* | no difference |  |  |
| *TSPAN8* | decreased | 0.007 | Richardson *et al.* |
| *TXNRD* | increased | 8.04x10^-4^ | Perou *et al.*  Sørlie *et al.* (2001)  Sørlie *et al.* (2003)  Zhao *et al.* |
| *UBE2N* | no difference |  |  |
| *USP44* | no difference |  |  |
| *UTP20* | increased | 0.001 | Richardson *et al.* |
| *VEZT* | increased |  |  |
| *ZDHHC17* | decreased | 1.51x10^-5^ | Richardson *et al.*  Sørlie *et al.* (2001)  Sørlie *et al.* (2003)  Zhao *et al.* |
| ^1^A p-value for *SLC6A15* was significant for decreased and increased level of *SLC6A15* depending on which study was considered.  Genes were compared across seven studies using Oncomine 4.4 Research Edition ([www.oncomine.org/resource/login.html](http://www.oncomine.org/resource/login.html)).  Only studies that included non-diseased breast tissue and ductal/invasive breast carcinomas were queried [S1-S7].  nr is used to indicate that a gene was not reported in any of the studies queried. | | | |
