# Supplementary material for: Physical Confirmation and Mapping of Overlapping Rat Mammary Carcinoma Susceptibility QTLs, Mcs2 and Mcs6
Source: PLoS One. 2011 May 18;6(5):e19891. doi: 10.1371/journal.pone.0019891 (PMC3097214; doi:10.1371/journal.pone.0019891)
Supplement: Table S2 — Human polymorphisms located in the Mcs6 orthologous region that have not reached genome-wide significance, but are potentially associated with breast cancer susceptibility. (DOCX) [file pone.0019891.s002.docx]

| **Table S2. Human SNPs in the *Mcs6* Orthologous Region that Have Been Reported in Genome-Wide Association Studies as Potentially Associating with Breast Cancer Susceptibility.** | | | | | |
| --- | --- | --- | --- | --- | --- |
| **SNP ID** | **Human Chr Location** | **Rat Chr Location** | **OR** | **P-value** | **Study** |
| *Rs4146372* | *12q72131594* | *7q54588907* | nr | **7.0 x 10^-5^** | Murabito *et al.* [S8] |
| *Rs1154865* | *12q 72276104* | *7q54476579* | nr | **6.6 x 10^-7^** | Murabito *et al.* [S8] |
| *Rs17740709* | *12q83423340* | *7q*[*43765270*](http://genome.ucsc.edu/cgi-bin/hgTracks?db=rn4&position=chr7:43765270-43765270) | 0.91 | 0.002 | Thomas *et al.* [S9] |
| *Rs7310517* | *12q89149235* | *7q37548015* | nr | 1.04x10^-3^ | Antoniou *et al.* [S10] |
| *Rs10507088* | *12q97879744* | *7q29148172* | nr | 5.12x10^-4^ | Antoniou *et al.* [S10] |
| Chr, chromosome; OR, odds ratio; nr, not reported | | | | | |
