# Supplementary material for: Physical Confirmation and Mapping of Overlapping Rat Mammary Carcinoma Susceptibility QTLs, Mcs2 and Mcs6
Source: PLoS One. 2011 May 18;6(5):e19891. doi: 10.1371/journal.pone.0019891 (PMC3097214; doi:10.1371/journal.pone.0019891)
Supplement: References S1 — Citations for supplemental references. (DOCX) [file pone.0019891.s003.docx]

1. Perou CM, Sørlie T, Eisen MB, van de Rijn M, Jeffrey SS, et al. (2000) Molecular portraits of human breast tumours. Nature 406: 747-752.
2. Radvanyi L, Singh-Sandhu D, Gallichan S, Lovitt C, Pedyczak A, et al. (2005) The gene associated with trichorhinophalangeal syndrome in humans is overexpressed in breast cancer. Proceedings of the National Academy of Sciences of the United States of America 102: 11005-11010.
3. Richardson AL, Wang ZC, De Nicolo A, Lu X, Brown M, et al. (2006) X chromosomal abnormalities in basal-like human breast cancer. Cancer Cell 9: 121-132.
4. Sørlie T, Perou CM, Tibshirani R, Aas T, Geisler S, et al. (2001) Gene expression patterns of breast carcinomas distinguish tumor subclasses with clinical implications. Proceedings of the National Academy of Sciences 98: 10869-10874.
5. Sørlie T, Tibshirani R, Parker J, Hastie T, Marron JS, et al. (2003) Repeated observation of breast tumor subtypes in independent gene expression data sets. Proceedings of the National Academy of Sciences of the United States of America 100: 8418-8423.
6. Turashvili G, Bouchal J, Baumforth K, Wei W, Dziechciarkova M, et al. (2007) Novel markers for differentiation of lobular and ductal invasive breast carcinomas by laser microdissection and microarray analysis. BMC Cancer 7: 55.
7. Zhao H, Langerod A, Ji Y, Nowels KW, Nesland JM, et al. (2004) Different Gene Expression Patterns in Invasive Lobular and Ductal Carcinomas of the Breast. Mol Biol Cell 15: 2523-2536.
8. Murabito J, Rosenberg C, Finger D, Kreger B, Levy D, et al. (2007) A genome-wide association study of breast and prostate cancer in the NHLBI's Framingham Heart Study. BMC Medical Genetics 8: S6.
9. Thomas G, Jacobs KB, Kraft P, Yeager M, Wacholder S, et al. (2009) A multistage genome-wide association study in breast cancer identifies two new risk alleles at 1p11.2 and 14q24.1 (RAD51L1). Nat Genet 41: 579-584.
10. Antoniou AC, Wang X, Fredericksen ZS, McGuffog L, Tarrell R, et al. (2010) A locus on 19p13 modifies risk of breast cancer in BRCA1 mutation carriers and is associated with hormone receptor-negative breast cancer in the general population. Nat Genet 42: 885-892.
11. Li J, Humphreys K, Darabi H, Rosin G, Hannelius U, et al. (2010) A genome-wide association scan on estrogen receptor-negative breast cancer. Breast Cancer Research 12: R93.
12. Turnbull C, Ahmed S, Morrison J, Pernet D, Renwick A, et al. (2010) Genome-wide association study identifies five new breast cancer susceptibility loci. Nat Genet 42: 504-507.
13. Garcia-Closas M, Hall P, Nevanlinna H, Pooley K, Morrison J, et al. (2008) Heterogeneity of Breast Cancer Associations with Five Susceptibility Loci by Clinical and Pathological Characteristics. PLoS Genet 4: e1000054.
14. Antoniou AC, Sinilnikova OM, McGuffog L, Healey S, Nevanlinna H, et al. (2009) Common variants in LSP1, 2q35 and 8q24 and breast cancer risk for BRCA1 and BRCA2 mutation carriers. Human Molecular Genetics 18: 4442-4456.
15. Stacey SN, Manolescu A, Sulem P, Thorlacius S, Gudjonsson SA, et al. (2008) Common variants on chromosome 5p12 confer susceptibility to estrogen receptor-positive breast cancer. Nat Genet 40: 703-706.
16. Stacey SN, Manolescu A, Sulem P, Rafnar T, Gudmundsson J, et al. (2007) Common variants on chromosomes 2q35 and 16q12 confer susceptibility to estrogen receptor-positive breast cancer. Nat Genet 39: 865-869.
17. Mavaddat N, Dunning AM, Ponder BAJ, Easton DF, Pharoah PD (2009) Common Genetic Variation in Candidate Genes and Susceptibility to Subtypes of Breast Cancer. Cancer Epidemiology Biomarkers & Prevention 18: 255-259.
18. Gold B, Kirchhoff T, Stefanov S, Lautenberger J, Viale A, et al. (2008) Genome-wide association study provides evidence for a breast cancer risk locus at 6q22.33. Proceedings of the National Academy of Sciences 105: 4340-4345.
19. Zheng W, Long J, Gao Y-T, Li C, Zheng Y, et al. (2009) Genome-wide association study identifies a new breast cancer susceptibility locus at 6q25.1. Nat Genet 41: 324-328.
20. Ahmed S, Thomas G, Ghoussaini M, Healey CS, Humphreys MK, et al. (2009) Newly discovered breast cancer susceptibility loci on 3p24 and 17q23.2. Nat Genet 41: 585-590.
21. Hunter DJ, Kraft P, Jacobs KB, Cox DG, Yeager M, et al. (2007) A genome-wide association study identifies alleles in FGFR2 associated with risk of sporadic postmenopausal breast cancer. Nat Genet 39: 870-874.
22. Rahman N, Seal S, Thompson D, Kelly P, Renwick A, et al. (2007) PALB2, which encodes a BRCA2-interacting protein, is a breast cancer susceptibility gene. Nat Genet 39: 165-167.
23. Easton DF, Pooley KA, Dunning AM, Pharoah PDP, Thompson D, et al. (2007) Genome-wide association study identifies novel breast cancer susceptibility loci. Nature 447: 1087-1093.
